# Would Moving Forward Mean Going Back? Comment on Maselli et al. Direct Access to Physical Therapy: Should Italy Move Forward? *Int. J. Environ. Res. Public Health* 2022, *19*, 555

**DOI:** 10.3390/ijerph19084579

**Published:** 2022-04-11

**Authors:** Antimo Moretti, Massimo Costa, Giovanna Beretta

**Affiliations:** 1 Department of Medical and Surgical Specialties and Dentistry, University of Campania “Luigi Vanvitelli”, 80138 Naples, Italy; 2 Physical and Rehabilitation Unit, AORN Vincenzo Cardarelli, 80131 Napoli, Italy; massimo.costa@aocardarelli.it; 3 Unit of Rehabilitation Medicine and Neurorehabilitation, Department of Neuroscience, ASST Niguarda Hospital, 20162 Milan, Italy; giovanna.beretta@ospedaleniguarda.it

In a recent communication, Maselli and colleagues [1] supported the direct access to physical therapy (PT) for patients with musculoskeletal disorders (MSKD), providing questionable reasons for the implementation of this model of care.

These authors based their proposal on two studies. One is a randomized controlled trial (RCT) carried out in an academic emergency department (ED) in Canada, which randomized 78 people who accessed the ED for a minor MSKD. One group was evaluated by a physical therapist following a nurse triage under the supervision of an ED physician, and patients of the other group were evaluated by the ED physician [2]. Patients of both groups were referred to the PT if necessary and were followed up after 3 months with no significant clinical differences found between groups [2]. The second study cited by the Authors aimed only to investigate the feasibility of a RCT on this topic [3].

Regardless of all of the biases related to the cited papers, it should be noted that they both analyzed populations from countries supporting the advocacy for direct access to PT, not for clinical reasons but for political and economic convenience [4,5]. Additionally, the other studies cited by Maselli et al. address costs instead of the safety and/or efficacy of direct access to PT compared with the physician-led approach (i.e., general-practioner-based, not medical-specialist-based). Moreover, the authors committed a misinterpretation by claiming a “higher level of confidence and appropriateness of physical therapists in the management of MSKD than physicians”. Indeed, Childs et al. [6] (cited by Maselli et al.) reported that the level of knowledge of physiotherapists was still lower than that of orthopedists, the only category of medical specialists with expertise in MSKD included in the study.

As for the issue regarding the areas of autonomy of the physical therapists, in Italy, their involvement in prevention, care, and rehabilitation activities for people with disabilities, is always secondary to both the diagnoses and prescriptions performed by the physicians, including in private practices [7]. It should also be noted that the Italian Law 251/2000, cited in the paper, allows for the functional evaluation by a physical therapist, whereas the diagnosis remains the responsibility of a physician [8].

Concerning “Figure 1”, presented by the authors in favor of the direct access to PT, there are two conceptual/practical mistakes: the first mistake is in the referral to a medical specialist (i.e., secondary care referral) for “further investigation (e.g., imaging)”, which is not the routine (see the guidelines for the management of low back pain, LBP) [9,10]. The second mistake concerns the savings in “secondary care setting treatments (e.g., medication)”, considering that drugs, if prescribed according to appropriateness criteria (i.e., in accordance with guidelines), do not represent a barrier but rather a facilitator for the functioning and general wellbeing of patients (particularly for those with MSKD), as well as in terms of cost effectiveness.

In our opinion, the most limiting aspect of the paper of Maselli et al. is the support of a dated “one-man show” approach (physical therapist only), which contrasts with the modern interdisciplinary and multimodal approach (see international guidelines for the management of LBP and osteoarthritis, OA) [9,10,11,12]. According to the above-mentioned guidelines, the best management strategy for people requiring rehabilitation is the biopsychosocial approach [13]. Indeed, physiotherapy is just one of the rehabilitation interventions (which includes a prescription of physical agents and therapeutic exercises) to be integrated with drug therapy and/or the use of assistive devices, psychological, behavioral, and cognitive interventions.

Finally, we want to remind these authors that the concept of multidisciplinary rehabilitation approach is the basis of strengthening rehabilitation all over the world, as claimed by the WHO Rehabilitation 2030, a call for action [14].

## Data Availability

Not applicable.
